# Paediatric Auto Renal transplantation-Anaesthetic Challenge

**Published:** 2009-08

**Authors:** Saravanan PA, Rebecca Jacob, Raj Sahajanandan, Anita Shirley Joselyn

**Affiliations:** 1,3,4 Asst Professor, Department of Anaesthesia and critical care, Christian Medical College & Hospital, Vellore, India-632004; 2 Professor, Department of Anaesthesia and critical care, Christian Medical College & Hospital, Vellore, India-632004

**Keywords:** Takayasu's arteritis, Paediatric, Auto renal transplantation, Anaesthesia

## Abstract

**Summary:**

Takayasu's arteritis is described to be the single most important cause of renovascular hypertension. Anaesthetising a child with Takayasu's arteritis for auto renal transplantation is a challenge as it is complicated by severe uncontrolled hypertension, end-organ dysfunction resulting from hypertension, stenosis of major blood vessels affecting regional circulation, and difficulties encountered in monitoring arterial blood pressure. A balanced anaesthetic technique, maintenance of stable haemodynamics with monitoring is required for a successful outcome. We describe the anaesthetic management of a child with Takayasu's arteritis and severe hypertension refractory to medical treatment requiring auto renal transplantation.

## Introduction

Severe persistent hypertension in childhood is mainly due to renalparenchymal disease with renovascular lesions being the second most common cause. Takayasu's arteritis is the single most important cause of renovascular hypertension in non white children^1^. Takayasu's arteritis is characterized by a focal stenosis process involving the aorta and the proximal segment of its major branches^2^. We present the anaesthetic management of a child with Takayasu's arteritis and severe hypertension refractory to medical treatment requiring auto renal transplantation.

## Case report

**Fig 1:**
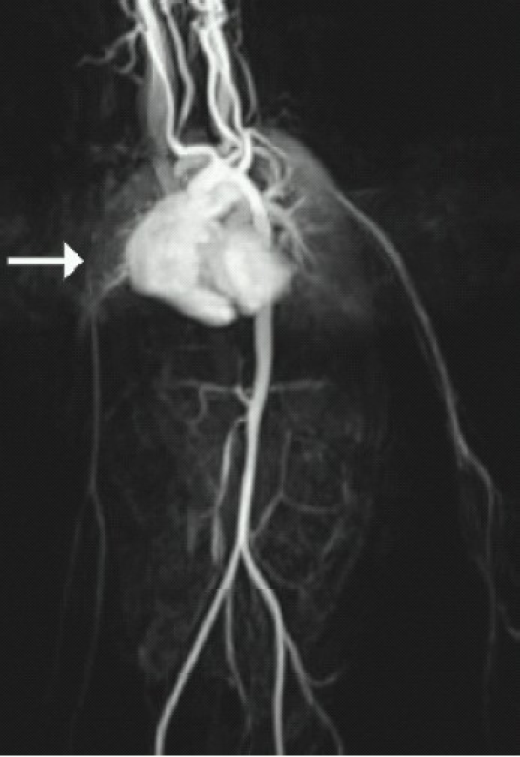
Aortography revealing occlusion of right subclavian artery. Left renal artery not visualized due to 99% stenosis on left side.

A 4-year-old male child weighing 13 kg, presented with history of dyspnoea on exertion for 3 month duration. On examination, all peripheral pulses were palpable except on the right upper limb. His blood pressure was 140/100 mm Hg in the left upper limb and was not recordable on the right upper limb. He was evaluated for secondary hypertension and found to have Type III Takayasu's arteritis with aortography revealing 99% stenosis of left main renal artery and 60% stenosis of the right main renal artery as well as occlusion of the right subclavian artery (Fig 1). He had clinical and echocardiographic evidence of left ventricular dysfunction with normal renal functions. He was stabilized on tab.nifedepine 5mg Q4H, prazosin 1mg Q6H, aldomet 250mg Q8H, minoxidil 5mg Q12H, frusemide 10 mg, digoxin 0.25 mg and aspirin once a day. He underwent balloon angioplasty and stenting of left renal artery. He presented subsequently with a history of recurrent episodes of seizures and persistently elevated blood pressure. MRI brain revealed vasculitis induced multiple infarcts. Right auto renal transplantation was considered due to refractory hypertension and ongoing complication. Preoperative vitals revealed GCS of 14/15, heart rate of 96/min, absent right radial pulse with blood pressure in left upper limb of 170/110mmHg. His blood investigations were normal with serum creatinine of 0.9mg%. Chest roentgenogram revealed cardiomegaly and electrocardiogram left ventricular hypertrophy.

**Fig 2:**
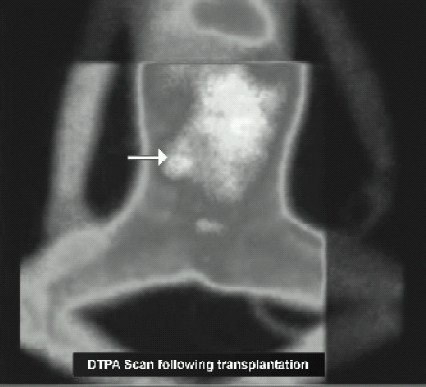
DTPA scan revealing uptake by auto transplanted kidney on right side.

**Fig 3:**
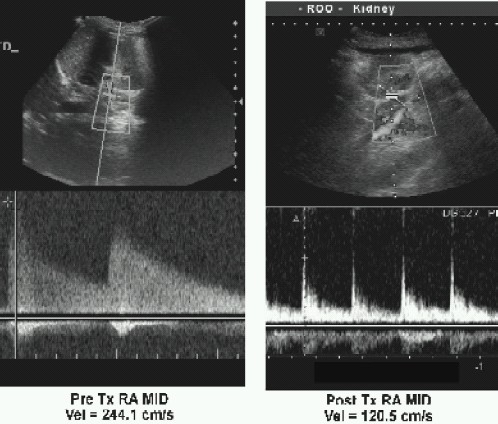
Colour doppler of right renalartery- pre and post renal autotransplantation

The patient was fasted and premedicated with Syp.Triclofos 75mg.kg^−1^. Under standard monitoring (SpO_2_, non-invasive blood pressure, ECG, end-tidal CO_2_ monitor, temperature), anaesthesia was induced with air, oxygen and sevoflurane. The trachea was intubated following atracurium administration and the patient mechanically ventilated. Under ultrasound guidance, a 5F triple lumen catheter was sited in the right internal jugular vein. The left femoral artery was selected for direct arterial pressure monitoring. Anaesthesia was maintained with 50% mixture of air and oxygen, end-tidal isoflurane concentration of 1% with morphine, fentanyl and atracurium boluses as needed. Normocarbia was maintained to preserve cerebral perfusion. Surgery was done in supine position. A good perfusion pressure of the transplanted kidney was ensured by maintaining a systolic blood pressure of 130 mm Hg, CVP of 13-15 mmHg. Mannito 10.5g.kg^−1^ was infused 20 minutes before clamp release. Blood loss was replaced with fresh whole blood. Urine output was more than 0.5 ml.kg^−1^.hr^−1^ after the anastamosis. The intraoperative period was uneventful. Arterialblood gas analysis was within normallimits and trachea extubated at the end of the procedure. Post operatively, the child was monitored in the high dependency unit and had an unremarkable stay. Outpatient visit at 8 weeks revealed improved sensorium and blood pressure of 130/85mmHg in left upperlimb. Diethylene Tetramine Penta Acetate(DTPA) scan done at 8 weeks showed functioning of auto transplanted kidney (Fig 2) and antihypertensives were tapered. Colour doppler revealed good perfusion of transplanted kidney as characterized by the reduction in the peaksystolic velocity from 244.1 cm/sec to 120.5 cm/sec. (Fig 3)

## Discussion

Patients with Takayasu's arteritis (TA) present for surgery either incidentally or more frequently for correction of the consequences of vascular occlusive disease. Anaesthetising such a child for auto renal transplantation is a challenge as it is complicated by severe uncontrolled hypertension, end-organ dysfunction resulting from hypertension, stenosis of major blood vessels affecting regional circulation, and difficulties encountered in monitoring arterial blood pressure^3^. Surgery is indicated in patients with uncontrolled hypertension, deteriorating renal function, lesions not amenable to angioplasty and transient response to angioplasty^4^. In our patient, auto renal transplantation was resorted to as he was not responding to antihypertensives and stenting of the stenosed left renal artery. Regional analgesia with epidural was not initiated in our patient as the risk of decrease in blood pressure may be hazardous in a patient with compromised regional circulation because of stenosed arteries^5^,^6^. This haemodynamic instability can also result in reduced graft function^7^. We used isoflurane to maintain the blood pressure at basal preoperative levels, decrease the cerebral metabolic rate of oxygen and produce cerebral vasodilatation.

Sudden hypertensive or hypotensive episodes are avoided intraoperatively so as to prevent possible cerebrovascular or cardiovascular crises^1^. Invasive arterial and venous pressure monitoring is considered mandatory as excessive hemodynamic variations could have serious consequences for regional organ perfusion. However, pulmonary artery catheterization was not performed in view of the potential risks exceeding the possible benefits in a small child^1^,^3^. The factors affecting the transplanted kidney's viability are intravascular volume and perfusion pressure. Adequate intravascular volume is the single most important determinant of immediate graft function. ACVP of 12- 14 mmHg is recommended to maintain optimal intravascular volume^8^. Acute tubular necrosis can result from inadequate hydration and leads to delayed graft function, decreased graft survival and increased patient morbidity. Mannitol when combined with volume expansion has been shown to decrease the incidence of acute tubular necrosis after transplantation^9^. Routine use of inotropes is not warranted and vasopressors should be given as the last resort^3^. Unexplained hypotension and inadequate response to inotropes may be due to underlying metabolic acidosis and should be corrected. These patients warrant intensive care stay in post operative period. Our patient had stable haemodynamics during the intraoperative period probably due to a balanced anaesthetic technique and CVP guided volume replacement.

Anaesthetising a child with Takayasu's arteritis for an auto renal transplantation is a challenge due to the various pathological and hemodynamic changes involved. Successful outcome depends on preoperative optimisation, intra operative maintenance of stable hemodynamics and invasive monitoring during perioperative period.
